# The Antioxidative Impact of Dietary Vinegar and Rocket Salad on the Productivity, Serum Oxidation System, and Duodenal Histology of Chickens

**DOI:** 10.3390/ani11082277

**Published:** 2021-08-02

**Authors:** Karrar Imad Abdulsahib Al-Shammari, Justyna Batkowska

**Affiliations:** 1 Department of Animal Production Techniques, Technical College of Al-Musaib, Al-Furat Al-Awsat Technical University, Babylon 54003, Iraq; Karrar.Al-Shammari@atu.edu.iq; 2 Institute of Biological Basis of Animal Production, University of Life Sciences in Lublin, 13 Akademicka St., 20-950 Lublin, Poland

**Keywords:** oxidative stress, broiler chickens, acetic acid, *Eruca sativa*

## Abstract

**Simple Summary:**

Oxidative stress (OS) is detrimental to the production of reactive oxygen species. It affects poultry performance, health status, welfare, and meat and eggs quality. Many methods of reducing oxidative stress have been considered; however, due to the birds’ welfare and the consumers’ safety, natural substances (antioxidants) have mainly been taken into consideration. The current data show that feeding broiler chickens Ross 308 with either a dietary supplementation of vinegar (5 and 10 mL/kg of diet) or rocket salad (2 and 3 g/kg of diet) could mitigate the experimentally induced oxidative stress level. Positive changes were demonstrated by the improvement of the serum oxidation system, which led to increases in the birds’ productivity and carcass quality, and modulations of intestine histomorphological characteristics. Thus, vinegar and rocket salad might be promising natural alternatives for costly feed additives under the various types of stresses or acute environmental conditions.

**Abstract:**

The purpose of the study was to investigate the ameliorative effect of dietary rocket salad and apple cider vinegar on the oxidative stress (OS) status of broilers. Specifically, 720 Ross 308 chicks were divided into six groups: negative and positive controls (NC and PC with and without additives, Vi1 and V12 experimental groups fed with diets mixed with 5 and 10 mL of vinegar/kg, and Ro1 and Ro2 groups fed with diets mixed with 2 and 3 g of rocket salad/kg, respectively). The experimental groups Vi1 and Vi2 were fed with feed mixture moisturized with 5 and 10 mL of vinegar/kg, and Ro1 and Ro2 were fed with 2 and 3 g of rocket salad/kg, respectively. The birds’ productivity, oxidative serum parameters, and morphometric indices of the gastrointestinal tract were registered at 6 weeks of rearing. The vinegar or rocket salad additive had powerful potentials to significantly suppress (*p* ≤ 0.05) OS through improving the birds’ survivability, body weight gain, feed conversion ratio, and carcass yield. The highest villus height and villus height/crypt depth ratio of the duodenum were achieved by Vi2, Ro1-2, and NC (*p* ≤ 0.05). The villus surface area and muscular layer thickness were smallest in the PC, while they did not differ significantly in other groups (*p* > 0.05). Similar relationships were found in serum superoxide dismutase, glutathione peroxidase, hydroperoxide, and malondialdehyde; however, higher doses of both additives were more effective. It seems that liquid vinegar and rocket salad could have beneficial influences on the antioxidant status of birds.

## 1. Introduction

The detrimental effect of oxidative stress (OS) to the production of reactive oxygen species can be prevented or alleviated by natural substances that are called antioxidants. OS affects avian performance, health status, welfare, and final products’ quality (meat and eggs) [1,2]. Antioxidant material is produced naturally by ROS (reactive oxygen species) in oxygen-rich environments and is oxidized either endogenously (in vivo) or exogenously (in vitro), acting to decrease the rate of chemical reactions between ROS and important cellular elements such as proteins, DNA, or lipid membranes [3,4]. Based on this fact, there is a growing need to use selected antioxidants from various sources in proper forms to increase poultry production [2].

Vinegar is a flavoring condiment and acidifier made by alcoholic fermentation from diverse sugary and starchy materials, and it is widely used for cooking purposes. Acetic acid (CH_3_COOH) is the predominant ingredient in apple cider vinegar, which is characterized by its antimicrobial and anticoccidial activity [5,6] with immune stimulation and its improvement of the productive performance [7], and egg production and hatchability [8]. In addition, cider vinegar might contain malic acid and different types of flavonoids involving quercetin, camproforol, anthocyanin, catechins, cyanidin-3-glucoside, and apicotin [9]. Acetic acid is one of the short-chain fatty acids and beneficial additives in poultry diets because of its excellent chemical and physical characteristics of changing the intestinal pH, lowering the viscosity, and modulating the feed utilization in the avian gut [10,11], and improving the intestinal histomorphology in broilers [7]. It could also be used as a chemotherapeutic drug due to its ability to limit and lower the level of pathogens and toxins colonization in the avian gut [12] with the better promotion of nutrient digestibility [13].

Rocket salad (*Eruca sativa* Mill), commonly known as arugula, is one of the annual herbs of the *Brassicaceae* family. It is characterized by its pungent spicy flavor and taste, which can be used for cooking purposes fresh or after preparation in steam [14,15]. Regarding animal nutrition, rocket salad has been used as a feed supplement in diets, particularly because of its main contents of essential oils, proteins, and bioactive compounds such as glucosinolates and erucic acid [16,17], vitamins (C, A), and potassium, iron, and sulfur [18]. The most important glucosinolates contained in seeds, leaves, and roots of this herb are desulfo-glucoraphanin, desulfo-glucoerucin, desulfo-dimeric 4-mercaptobutyl glucosinolate, and desulfo-4-methoxyglucobrassicin [14], which serve the antioxidative properties and play multiple beneficial biological roles.

It was hypothesized that, due to their antioxidative properties, both rocket salad and vinegar may also be useful in oxidative stress reduction in poultry. However, according to our findings, there have been very limited reports about using rocket salad or vinegar mixed in poultry diets, especially concerning their antioxidative activity, particularly in poultry. Therefore, the aim of the current work was to unveil the possible antioxidative activity of rocket salad (*Eruca sativa* Mill.) supplemented in diets or using apple cider vinegar mixed with diets to alleviate the OS of broiler chickens fed with lead acetate as an oxidative promoter. The effect of this process on productive performance, gut histomorphology, and oxidation parameters of blood serum was investigated.

## 2. Materials and Methods

A total of 720 unsexed Ross 308 1-day-old broiler chicks were purchased from a local hatchery and raised in an environmentally controlled Poultry Farm affiliated with the Technical College of Al-Musaib, Al-Furat Al-Awsat Technical University, Babylon, Iraq. The chicks were individually marked by wing number and kept in a floor rearing system (2 m × 2 m pens). With regard to animal welfare and ethical principles, all experimental animal treatments were approved by the Scientific Committee of Department of Animal Production Techniques, Technical College of Al-Musaib Al-Furat Al-Awset Technical University (Babylon, Iraq).

Chicks were fed ad libitum on an isonitrogenous and isocaloric diet based on soybean and corn [19] with free access to water. The lighting program 6:18 D:L was maintained for the whole period from day 1 until six weeks of age. The diets’ composition is given in Table 1. Birds were divided into 6 groups, 120 chicks per group with 4 equal replications (pens). The groups of birds were distributed into the following: NC—negative control without exposition to OS and without any supplementary additives in diet; PC—positive control exposition only to 200 ppm of lead acetate/L of drinking water; Vi1 and Vi2—moisturization with 5 and 10 mL of vinegar/kg of diet, respectively; Ro1 and Ro2—adding 2 and 3 g of rocket salad/kg of diet, respectively. The same additive of PC (200 ppm of lead acetate/L of drinking water) was added to Vi1, Vi2, Ro1, and Ro2.

Powdery lead acetate (99% purity, Lab Tech Chemicals, Gunsheim-Gustavsgurg, Germany) was used as an oxidative factor to artificially induce OS in broilers by adding it daily to drinking water [20,21].

Apple cider vinegar and rocket salad used as antioxidant materials in diets were both commercially available and purchased from the local market. Apple cider vinegar (5% acetic acid) was in liquid form, which was manufactured by Food Canning Company, Karbala, Iraq. It was applied as an antioxidative material via moisturization with the diet by the mixing method of (volume:weight) either 5 or 10 mL per kg of diet. The dietary moisturization with vinegar was performed daily to preserve its pureness property and prevention from volatilization.

The powdery texture of rocket salad (*Eruca sativa* Mill.) was made from seeds of plants obtained from the commercial market. It was tightly packaged in a black polyethylene bag after mixing in feed by 2 and 3 g of rocket salad/kg. The chemical composition of rocket salad (Table 2) was determined in accordance with AOAC [22] based on dry weight. In addition, the detection and calculation of the bioactive compounds, including total phenols, flavonoids, glycosides, saponins, tannins, and alkaloids (Table 3), were performed by using the analytical methods described routinely by Zare et al. [23], Babaa and Malikba [24], Kaur and Arora [25], Anhawange et al. [26], Abdelkader et al. [27], and Ajanal et al. [28], respectively. All chemical analyses of bioactive compounds were carried out with the spectrophotometric method using HPLC (high-performance liquid chromatography, Shimadzu LC-2010CHT HPLC System, Shimadzu 2010, Tokyo, Japan).

For the whole period of experiment, the diet offered to birds was in crumble form from 1 until 6 weeks of the experiment to facilitate the mixing of the seeds of rocket salad powder in the feed and moisturization of the feed with vinegar daily. Thus, the rocket salad was prepared every 3 days.

The averages of productive characteristics (*n* = 4/treatment) were recorded weekly and presented accumulatively, including body weight, weight gain, feed intake, and feed conversion ratio (FCR). Water intake and survivability were also calculated. The production efficiency factor (PEF) as an indicator of the economic profit of trial was calculated based on Lemme et al. [29].

The slaughtering of 4 birds per replicate was carried out on the 6th week via cervical dislocation and then decapitation. The birds had limited access to feed for 8 h before the slaughter. The carcass yield and proportions of carcass cuts and abdominal fat out of live body weight were registered. Samples of the gastrointestinal tract were collected to analyze their morphometric parameters (*n* = 16/treatment). All sections of the gut starting from the esophagus to the rectum were measured using a vernier caliper and tape measure. To measure the crop, proventriculus, and gizzard length, a tape measure was used for this purpose, while the lengths of the duodenum, jejunum, ileum, ceca, and rectum sections were measured by a vernier caliper. The relative weights of all visceral organs (lungs, kidneys, adrenals, pancreas, spleen, bursa of Fabricius, and thymus gland) were calculated after registering the absolute weights using an accurate-sensitivity (0.01 g) digital scale (Aarson Digital Balance, Haryana, India). Then, 1 cm of duodenum (always the same part) was removed and placed in a plastic container containing 10% formalin for fixation and then dehydrated and embedded with paraffin. Later, a 4 μm thickness was taken and exposed to staining with hematoxylin and eosin to process the investigation of villi and crypts dimensions and muscular layer thickness. The histological analysis was performed from 4 birds per replicate of treatment with 2 sections per bird, and 10 well-oriented villi and crypts from the duodenum of each slide were measured (20 slides per bird). Then, the average value for each bird was used for further statistical analysis (*n* = 16/treatment). All methods of investigation were carried out according to the procedures reported by Rubio et al. [30] and Al-Shammari et al. [2].

Blood samples were placed in a gel separator tube after being collected from a neck vein during slaughtering (*n* = 16/treatment). To separate serum, blood samples were centrifuged for 15 min at 3000 RPM, and then the serum samples were preserved at −25 °C until analysis. Indicators of lipid peroxidation in serum involving malondialdehyde (MDA) and hydroperoxide (LOOH) were determined according to Salih et al. [31] and Södergren et al. [32], respectively. In addition, values of serum antioxidative enzymes such as catalase (CAT), superoxide dismutase (SOD), and glutathione peroxidase (GPx) were recorded based on the procedures mentioned by Aebi [33] and Misra and Fridovich [34]. With respect to the measurement of the ferric-reducing ability of plasma (FRAP), the analytical method of Benzie and Strain [35] was followed. All analyses in serum for oxidation parameters were carried out by using a spectrophotometer (BS 130 Mindray, Shenzhen Mindray Bio-medical Electronics Co., Shenzhen, China) with commercial kits (Sigma Aldrich, St. Louis, MO, USA). Each measurement was made twice.

A completely randomized design was used in analyzing the experimental data to find the effect of different groups on variables. The Shapiro–Wilk test was carried out in the case of normality of data. The significant differences among groups’ general means were compared by using the Duncan test [36] through application of the software program SAS [37] (2010) and on the basis of the following model:

Y_ij_ = µ + t_i_ + e_ij_ (1)

Y_ij_—studied trait (i) under effect of treatment (j);

µ—the general average of the studied trait;

t_i_—the effect of treatment;

e_ij_—random error.

## 3. Results

The data presented in Table 4 show that there were no significant differences in body weight at 3 weeks, as well as feed intake and water intake at 1–3 weeks; however, there were significant increases (*p* ≤ 0.05) in body weight at 6 weeks, weight gain and feed intake at 1–6 weeks, and survivability in favor of all experimental groups and NC compared to PC. All experimental groups also demonstrated a significant decrease (*p* ≤ 0.05) in feed conversion ratio at 1–3 weeks and 1–6 weeks compared to PC.

The production efficiency factor, carcass yield, and breast ratio were significantly higher (*p* ≤ 0.05) in experimental groups and NC (Table 5) with the decrease (*p* ≤ 0.05) in abdominal fat ratio in the same groups compared with PC. In comparison with PC, Vi1, Vi2, and Ro1 exhibited high thigh ratios (*p* ≤ 0.05), while Vi1, Vi2, Ro1, and Ro2 exhibited high drumstick ratios (*p* ≤ 0.05). However, increases in wings, back, and neck ratios were registered in PC compared to other groups.

It is clear from Table 6 that the lengths of the small and large intestines (%) and weights of the liver and gizzard (%) were high, as recorded by all experimental groups, whereas the weights of the small intestine (%) were found to be considerably higher in Vi2, Vi2, Ro1, and Ro2 compared to PC; similar values were demonstrated in samples from the NC group. The whole gut weight (%) was highest (*p* ≤ 0.05) in Ro2 in comparison to the group with induced oxidative stress. No significant differences among groups were found in other anatomical traits.

Regarding Table 7, the villus height increased (*p* ≤ 0.05) in Vi2, Ro1, Ro2, and NC, and there was an increase (*p* ≤ 0.05) in villus width, villus surface area, and muscular layer thickness in all experimental groups and NC compared to PC. The Vi2, Ro1, Ro2, and NC registered an increase (*p* ≤ 0.05) in villus height/crypt depth with a lack of differences among groups in crypt depth.

All experimental groups and NC experienced significant decreases (*p* ≤ 0.05) in blood serum LOOH and MDA levels, although in the same groups, the blood serum SOD, GPx, and FRAP were high (*p* ≤ 0.05) compared to in PC. Moreover, Vi1, Vi2, Ro1, and NC had a higher (*p* ≤ 0.05) blood serum CAT in relation to PC (Table 8).

## 4. Discussion

The improvement of productive characteristics of stressed birds in groups fed with a wet diet with vinegar might be related to the influence of acetic acid found in vinegar in lowering the pH, thus limiting pathogenic microorganism growth through shifting the composition of the microbiome [7,13] such as aerobic, anaerobic, and coliform bacteria [8] in avian gut. The reduction in fecal *Escherichia coli* and *Salmonella* spp. development [6,38] leads to the increase in nutrient utilization and improvement of the productive performance [10]. A similar result was obtained by Abbas et al. [5] who found that adding various levels (1, 2, and 3%) of acetic acid to drinking water as an anticoccidial factor against *Eimeria tenella* from days 10 to 19 increased weight gain through the reduction in the cecal pH on the third, fifth, and seventh days but without affecting the FCR and mortality of broiler chickens. In addition, acetic acid worked along with butyric acid, citric acid, or formic acid to improve digestibility through reducing the pH and digesta viscosity in the duodenum and decreasing fecal moisture in broilers [11]. Under challenging conditions of broilers by *Salmonella pullorum*, Saleem et al. [6] found that 1% acetic acid supplemented in the diet showed a protective influence demonstrated by the achievement of better growth performances and feed conversion ratios, which could result from decreases in the rates of gross and histopathological changes in the liver. A similar result to the current data for the NC group was obtained by Hayajneh [39], who mentioned that offering the solution of 1, 2, and 3% apple cider vinegar in drinking water from 1 to 7 weeks did not affect the feed intake or the body weight gain of broiler chickens; however, body weight and FCR were not affected either. In addition, it was concluded that feed intake, body weight gain, carcass traits, edible organs, proportions of breast meat, and leg and abdominal fat were not influenced by feeding broilers from 4 to 16 weeks with a 1% dietary mixture of bamboo charcoal powder and bamboo vinegar liquid containing 2.87% acetic acid [38]. Probably, in the case of our study, a higher dose of apple vinegar was effective, especially in avoiding the OS effect, which resulted in better productivity effects.

The rocket salad powder added in Ro1 and Ro2 also had positive effects on the productive performance and carcass characteristics under the OS condition, probably due to its nutritive value and bioactive compounds found in chemical analyses of the extracted seeds (Table 2 and Table 3). Under normal feeding conditions, the diet included up to 5% of extract, and it did not have any negative effect on body weight and weight gain; in addition, 15% of this extract in the diet did not change the feed intake of broilers [40]. The most important phenolic compounds present in rocket salad are various glucosinolates and erucic acid [14,16,17]. Glucosinolate exhibits diverse biological functions, including antioxidant, antifungal, anti-carcinogenic, and antibacterial influences with immune-stimulant properties [41]. Rocket salad has also been reported to improve the general health status of the body by decreasing the serum aspartate aminotransferase (AST) and alanine transaminase (ALT) and lipid profile in quail males and females [42]. However, in our results, there were no differences between NC and all feed additives with H_2_O_2_ in productive traits. Razooqi et al. [43] stated that adding 1000 mg of oil of rocket salad per kg of diet under nonstressed conditions resulted in improvements of final body weight, weight gain, and FCR in broilers. In accordance to our results, Shani et al. [44] found that offering 0.25% seed powder of rocket salad in the broilers diet could palliate the OS induced by 0.5% H_2_O_2_ in drinking water through clear improvements in body weight, weight gain, and FCR with the increase in the antibody titers against Newcastle and Gumboro disease viruses.

The improvement of intestinal morphology, such as increases in villi dimensions, muscular layer thickness, and villus height/crypt depth ratio with the increase in the proportional length and weight of the small intestine in both experimental groups, might have a positive effect on organic acids as powerful acidifiers. Organic acids act to augment the immunity of the gut, inhibit inflammatory intensity at the digestive mucosa, develop the function of enzymatic secretions, and facilitate the digestion and absorption of beneficial nutrients [45]. Most organic acids with simple mono-carboxylic acids, such as acetic acid, have multiple and various mechanisms in vivo, which might undergo neutralization by the secretion of bicarbonates in the first part of an animal intestine to be more available for the gut [46]. Our results are incompatible with those by Rattanawut [38] who stated that the villus height and villus area of jejunum were higher, compared to NC in birds fed with 1% bamboo vinegar liquid containing acetic acid, although the total intestinal length and weight were not affected significantly. In addition, our observation differs from the results by Rehman et al. [7], who concluded that the villus height, crypt depth, and villus surface area of the duodenum and intestinal length and weight were increased in unstressed broilers (control group) fed with various levels of acetic acid (0.1, 0.2, and 0.3%) in diet. These discrepancies could result from different properties of the wide range of natural vinegars resulting from their chemical composition. The effects of vinegar arose from consuming bioactive components including acetic acid, gallic acid, catechin, epicatechin, chlorogenic acid, caffeic acid, *p*-coumaric acid, and ferulic acid [47,48]. At the same time, it is impossible to point to the most effective substance.

There is a lack of studies pertaining to the effect of rocket salad on the anatomical and morphological traits of the avian intestine. However, the improvement in these characteristics in Ro1 and Ro2 might be due to the importance of powerful compounds found in rocket salad with their aromatic properties to stimulate the secretion of digestive enzymes, activate appetite, increase the beneficial modulation of microbes, and enhance the immune system of the gut [43,44]. These changes were reflected by improving the metabolism and absorption in the gut and enhancing intestinal morphology, productive traits, and carcass yield of birds during stressful times (Table 4, Table 5 and Table 6).

All the feed additives performed positive roles to maintain the oxidative status and suppress OS through boosting the antioxidative enzymes (SOD, CAT, and GPx) and FRAP while lowering lipid peroxidation markers (LOOH and MDA) in serum. The antioxidant enzymes are essential for modifying harmful oxygen molecules into nontoxic molecules, which inhibits the peroxidation of lipids [1]. The mechanism of acetic acid acting against oxidative stress is not clear yet. Probably it may be change to: However, the improvement in serum oxidation parameters in Vi1 and Vi2 might resulted from the acetic acid influence on the increase of digestibility and bioavailability of nutrients that are important in alleviating stress. This is obvious in the positive occurrence of intestinal functions such as the digestion, absorption, and metabolism of nutrients [8,10,11]. Saleh et al. [49] declared that there was an antioxidative improvement in the lipids’ peroxidation level, total phenolic amounts, and 1,1-diphenyl-2-picrylhydrazyl radical scavenging activity with the increase in long-chain polyunsaturated fatty acids (n-3) in the thigh meat of chickens fed with 200 mg of α-tocopherol acetate (ester of acetic acid and α-tocopherol) per kg of diet. The same data were obtained by Fouad et al. [8], who concluded that there was a protection effect from oxidative damage by adding 0.5 and 1 mL of vinegar/L of drinking water via the presence of escalating levels of antioxidative enzymes (GPX SOD) and glutathione in the serum of quails exposed to high-temperature stress during summer from 8 to 14 weeks. All these observations were confirmed in our research. Supplemented groups, regardless of substance, had similar values of serum oxidative markers to NC groups not exposed to OS, and higher values than PC exposed to oxidative stress factors. Increases in the oxidative stress markers in Ro1 and Ro2 are probably due to the presence of antioxidant substances in rocket salad, such as total phenols (Table 2), which participated in the reduction in the toxic influences in different mechanisms and removal of the deleterious impacts of oxidative factors [50,51]. This might also be the protective effect of flavonoid compounds in rocket salad against stress conditions and immune inhibition, which have important roles in mediating the boosting of inflammatory cytokines and counteracting the stress [44]. In addition, the putative antioxidative activity of rocket salad might be attributed to the active phenolic metabolites (glucosinolates) with their products (isothiocyanates, thiocyanates, and nitriles), which are pungent and bitter compounds. Each particular glucosinolate is a sulfur-containing compound containing asymmetrical S–S bonds important to the donation of electrons to free radicals and breaking down the hydroperoxides and H_2_O_2_ [14]. No data have documented the effect of rocket salad extract on OS condition and antioxidative indicators in poultry. However, El-Missiry et al. [52] found decreases in MDA and 4-hydroxynonenal in the liver with stimulation of the glutathione production by the oral intake of oil from rocket salad seeds in diabetic rats stressed by alloxan (0.06 mL/kg of body weight daily). In addition, the lowering of MDA in the liver was noticed, and elevated liver antioxidant enzymes including glutathione S-transferase (GST), SOD, and GPX resulted from giving rocket salad in four different forms (leaves and seed powders by 5% each instead of cellulose diet or in juice or oil forms by 300 mg or 3 mL/kg of body weight, respectively) in rats exposed to liver damage by paracetamol [53]. In ram lambs, the increase in serum total antioxidant capacity was recorded after 6 months of feeding with 2 mg of rocket salad oil during hot climate stress [50]. Under thermal stress conditions, it was found that 3% dietary rocket salad leaves enhanced liver SOD and CAT activities with a depression in MDA and cortisol levels in *Oreochromis niloticus* fish. At the same time, increases in genes expression, upregulation of the immune system, and antioxidation in fish liver exposed to OS through low and high temperature (18 and 32 °C, respectively) were noticed [51].

## 5. Conclusions

The current data prove that the feeding of broiler chickens Ross 308 with either a dietary moisturization with vinegar (5 and 10 mL/kg of diet) or dietary supplementation of rocket salad (2 and 3 g/kg of diet) could mitigate the oxidative stress induced experimentally by lead acetate in drinking water. Positive changes were demonstrated by the improvement of the serum oxidation system, which, in turn, led to increases in birds’ productivity and carcass quality, and the modulation of intestine histomorphological characteristics. Thus, vinegar and rocket salad might be natural promising alternatives for costly feed additives during the various types of stresses or acute environmental conditions.

## Figures and Tables

**Table 1:** The composition of birds’ diets.

Feed Stuffs	Starter (1 Day–3 Weeks)	Finisher (4 Weeks–6 Weeks)
Yellow corn	43.5	44.3
Wheat	18.0	18.0
Soybean	25.8	23.6
Protein concentrate	10.0	10.0
Salt	0.30	0.30
Limestone	0.40	0.40
Vegetable oil	2.00	3.40
Total	100	100
**Calculated chemical composition**		
Crude protein	22.11	21.20
Metabolizable energy (kcal/kg)	3105.6	3005.5
Calcium (%)	146.5	136.0
Lysine (%)	1.13	1.02
Methionine + Cystein (%)	0.79	0.65
Available phosphorus (%)	1.01	1.03
Crude fiber (%)	0.42	0.47
Ether extract (%)	3.461	3.600

**Table 2:** Chemical composition of powdered rocket salad seeds.

Component	(%)
Dry matter	95.22
Crude protein	28.21
Crude fiber	15.73
Ether extract	30.10
Nitrogen ether extract	17.24
Crude ash	7.91

**Table 3:** Bioactive compounds in powdered rocket salad seeds.

Bioactive Compound	(%)
Total phenols	26.97
Flavonoids	24.43
Glycosides	2.76
Saponins	6.20
Tannins	4.15
Alkaloids	11.27

**Table 4:** Productive traits of broiler chickens affected by dietary vinegar and rocket salad of stressed broiler chickens (*n* = 4/treatment).

Variables	Age of Birds (Weeks)	Groups						SEM	*p*-Value (F Test)
		NC	PC	Vi1	Vi2	Ro1	Ro2		
body weight (g)	1–3	813.5	699.5	732.5	818.00	775.01	807.5 ± 0	9.217	0.068
	1–6	2675.5 ^b^	1774.5 ^c^	2738 ^ab^	2830.5 ^a^	2819.5 ^a^	2749 ^a^	14.478	0.036
total weight gain (g)	1–3	771.15 ^a^	657.45 ^b^	690.17 ^b^	775.96 ^a^	731.87 ^ab^	766.59 ^a^	11.136	0.012
	1–6	2633.42 ^ab^	1732.45 ^c^	2696.67 ^ab^	2788.46 ^a^	2776.37	2707.09 ^a^	17.640	0.011
feed intake (g/day)	1–3	931.0	894.5	851.5	883.0	894.5	861.0	14.729	0.071
	1–6	3908.5 ^a^	3000.5 ^b^	3999.0 ^a^	3957.0 ^a^	3779.5 ^a^	3911 ^a^	18.262	0.041
feed conversion ratio (kg/kg of body weight gain)	1–3	1.20 ^b^	1.36 ^a^	1.23 b	1.13 ^c^	1.22 ^b^	1.12 ^c^	0.027	
	1–6	1.55 ^b^	1.75 ^a^	1.48 ^c^	1.41 ^c^	1.36 ^c^	1.44 ^c^	0.028	0.000
water intake (mL/day)	1–3	612.0	450	540.5	527.0	558.0	526.5	13.479	0.061
	1–6	1210.0 ^a^	990 ^b^	1242.5 ^a^	1253.5 ^a^	1305.5^a^	1234.5 ^a^	22.134	0.058
survivability (%)	1–6	100.0 ^a^	87.5 ^c^	97.5 ^b^	97.5 ^b^	100.0 ^a^	100.0 ^a^	4.079	0.043

NC: negative control; PC: 200 ppm of lead acetate/L of drinking water (positive control); Vi1 and Vi2: 200 ppm of lead acetate/L of drinking water + 5 or 10 mL of vinegar/kg of diet, respectively; Ro1 and Ro2: 200 ppm of lead acetate/L of drinking water + 2 or 3 g of rocket salad/kg of diet, respectively; SEM—standard error of mean, ^a,b,c^—means within rows with different superscripts differ significantly at *p* ≤ 0.05.

**Table 5:** PEF, carcass yield, and abdominal fat (%) of broiler chickens affected by dietary vinegar and rocket salad of stressed broiler chickens (*n* = 16/treatment).

Variables		Groups						SEM	*p*-Value (F Test)
		NC	PC	Vi1	Vi2	Ro1	Ro2		
PEF		410.97 ^a^	210.80 ^b^	427.95 ^a^	462.90 ^a^	490.67 ^a^	452.16 ^a^	18.58	0.000
carcass yield (%)		75.87 ^a^	72.17 ^b^	76.34 ^a^	75.65 ^a^	73.32 ^b^	74.76 ^a^	4.08	0.039
carcass cuts (%)	breast muscles	27.25 ^a^	24.55 ^b^	28.63 ^a^	27.35 ^a^	27.25 ^a^	27.64 ^a^	0.82	0.014
	thighs	18.76 ^ab^	17.66 ^b^	19.54 ^a^	19.84 ^a^	19.76 ^a^	18.86 ^ab^	0.85	0.043
	drumsticks	16.87 ^ab^	15.87 ^b^	17.26 ^a^	17.58 ^a^	16.77 ^a^	16.86 ^a^	1.87	0.036
	wings	11.76 ^b^	13.76 ^a^	11.52 ^b^	11.46 ^b^	11.72 ^b^	11.96 ^b^	1.69	0.012
	back	17.87 ^b^	19.67 ^a^	17.23 ^b^	17.47 ^b^	17.82 ^b^	17.73 ^b^	0.84	0.041
	neck	6.98 ^b^	7.98 ^a^	6.42 ^b^	6.29 ^b^	6.78 ^b^	6.93 ^b^	0.62	0.017
abdominal fat (%)		0.34 ^b^	1.85 ^a^	0.12 ^b^	0.48 ^b^	0.39 ^b^	0.43 ^b^	0.01	0.000

NC: negative control; PC: 200 ppm of lead acetate/L of drinking water (positive control); Vi1 and Vi2: 200 ppm of lead acetate/L of drinking water + 5 or 10 mL of vinegar/kg of diet, respectively; Ro1 and Ro2: 200 ppm of lead acetate/L of drinking water + 2 or 3 g of rocket salad/kg of diet, respectively; SEM—standard error of mean, ^a,b^—means within rows with different superscripts differ significantly at *p* ≤ 0.05. PEF—production efficiency factor.

**Table 6:** Visceral organs’ relative weight and length (as a proportion of total gastrointestinal tract weight or length, respectively) affected by dietary vinegar and rocket salad of stressed broiler chickens (*n* = 16/treatment).

Variables		Groups						SEM	*p*-Value (F Test)
		NC	PC	Vi1	Vi2	Ro1	Ro2		
small intestine	Length (%)	8.01 ^a^	6.01 ^b^	8.11 ^a^	8.35 ^a^	8.25 ^a^	8.87 ^a^	0.674	0.003
	weight (%)	5.18 ^a^	3.15 ^b^	4.26 ^ab^	5.53 ^a^	5.84 ^a^	5.42 ^a^	0.330	0.016
large intestine	length (%)	2.26 ^a^	1.06 ^b^	2.35 ^a^	2.42 ^a^	2.18 ^a^	2.58 ^a^	0.140	0.001
	weight (%)	0.84	0.73	0.79	0.81	0.80	0.89	0.003	0.074
whole gut weight (%)		9.27 ^ab^	8.13 ^b^	9.38 ^ab^	9.19 ^ab^	9.32 ^ab^	9.52 ^a^	0.385	0.045
giblets weight	liver (%)	2.89 ^a^	1.98 ^b^	2.98 ^a^	2.83 ^a^	2.76 ^a^	2.99 ^a^	0.053	0.021
	gizzard (%)	2.43 ^a^	1.65 ^b^	2.15 ^a^	2.23 ^a^	2.18 ^a^	2.12 ^a^	0.089	0.017
	heart (%)	1.18	1.02	1.36	1.19	1.18	1.29	0.095	0.800
lungs weight (%)		0.74	0.61	0.72	0.77	0.75	0.66	0.008	0.680
kidneys weight (%)		0.51	0.47	0.53	0.49	0.48	0.54	0.008	0.712
adrenals weight (%)		0.02	0.01	0.03	0.02	0.03	0.03	0.001	0.590
pancreas weight (%)		0.23	0.20	0.24	0.22	0.25	0.26	0.003	0.808
spleen weight (%)		0.21	0.19	0.22	0.22	0.23	0.21	0.003	0.921
bursa of Fabricius weight (%)		0.16	0.13	0.15	0.16	0.15	0.14	0.005	0.195
thymus gland weight (%)		0.12	0.11	0.12	0.11	0.12	0.11	0.000	0.950

NC: negative control; PC: 200 ppm of lead acetate/L of drinking water (positive control); Vi1 and Vi2: 200 ppm of lead acetate/L of drinking water + 5 or 10 mL of vinegar/kg of diet, respectively; Ro1 and Ro2: 200 ppm of lead acetate/L of drinking water + 2 or 3 g of rocket salad/kg of diet, respectively; SEM—standard error of mean, ^a,b^—means within rows with different superscripts differ significantly at *p* ≤ 0.05.

**Table 7:** Duodenal histomorphology affected by dietary vinegar and rocket salad of stressed broiler chickens (*n* = 16/treatment).

Variables		Groups						SEM	*p*-Value (F Test)
		NC	PC	Vi1	Vi2	Ro1	Ro2		
villus	height (μm)	1320.0 ^a^	920.0 ^c^	1110.0 ^bc^	1365.0 ^a^	1254.0 ^ab^	1325.0 ^a^	43.61	0.000
	width (μm)	184.0 ^a^	128.0 ^b^	178.0 ^a^	182.0 ^a^	177.0 ^a^	183.0 ^a^	11.68	0.001
	surface area (×10^3^ μm^2^)	762.13 ^a^	368.75 ^c^	621.61 ^ab^	781.66 ^a^	695.40 ^ab^	761.14 ^a^	21.36	0.000
crypt depth (μm)		183.11	170.14	177.31	176.25	189.21	186.42	18.03	0.874
villus height/crypt depth		7.31 ^a^	5.31 ^b^	6.47 ^ab^	7.85 ^a^	6.73 ^a^	7.42 ^a^	0.89	0.011
muscular layer thickness (μm)		276.12 ^a^	199.23 ^b^	269.43 ^a^	279.52 ^a^	253.32 ^a^	265.32 ^a^	15.57	0.007

NC: negative control; PC: 200 ppm of lead acetate/L of drinking water (positive control); Vi1 and Vi2: 200 ppm of lead acetate/L of drinking water + 5 or 10 mL of vinegar/kg of diet, respectively; Ro1 and Ro2: 200 ppm of lead acetate/L of drinking water + 2 or 3 g of rocket salad/kg of diet, respectively; SEM—standard error of mean, ^a,b,c^—means within rows with different superscripts differ significantly at *p* ≤ 0.05.

**Table 8:** Serum oxidation indicators affected by dietary vinegar and rocket salad of stressed broiler chickens (*n* = 16/treatment).

Variables	Groups						SEM	*p*-Value (F Test)
	NC	PC	Vi1	Vi2	Ro1	Ro2		
hydroperoxide (LOOH, μmol/L)	12.53 ^c^	18.42 ^a^	15.97 ^b^	12.31 ^c^	13.23 ^c^	12.52 ^c^	1.54	0.011
malondialdehyde (MDA, μmol/L)	10.31 ^c^	17.27 ^a^	11.32 ^c^	13.32 ^b^	10.35 ^c^	11.35 ^c^	1.78	0.000
superoxide dismutase (SOD, U/mL)	123.9 ^a^	95.34 ^c^	109.8 ^b^	124.9 ^a^	116.9 ^b^	124.3 ^a^	7.36	0.000
catalase (CAT, U/mL)	6.41 ^a^	4.63 ^b^	6.45 ^a^	7.44 ^a^	6.71 ^a^	5.67 ^ab^	0.72	0.003
glutathione peroxidase (GPx, U/L)	1.82 ^a^	1.23 ^b^	1.68 ^a^	1.92 ^a^	1.81 ^a^	1.76 ^a^	0.10	0.023
ferric-reducing ability of plasma (FRAP, μmol/L)	725.2 ^ab^	659.3 ^c^	852.2 ^a^	732.7 ^ab^	802.1 ^a^	721.2 ^ab^	12.19	0.017

NC: negative control; PC: 200 ppm of lead acetate/L of drinking water (positive control); Vi1 and Vi2: 200 ppm of lead acetate/L of drinking water + 5 or 10 mL of vinegar/kg of diet, respectively; Ro1 and Ro2: 200 ppm of lead acetate/L of drinking water + 2 or 3 g of rocket salad/kg of diet, respectively, SEM—standard error of mean, ^a,b,c^—means within rows with different superscripts differ significantly at *p* ≤ 0.05.

## Data Availability

The data presented in this study are available on request from the corresponding author.
